# Nucleotide excision repair of abasic DNA lesions

**DOI:** 10.1093/nar/gkz558

**Published:** 2019-06-21

**Authors:** Nataliya Kitsera, Marta Rodriguez-Alvarez, Steffen Emmert, Thomas Carell, Andriy Khobta

**Affiliations:** 1 Unit “Responses to DNA Lesions”, Institute of Toxicology, University Medical Center of the Johannes Gutenberg University Mainz, Mainz 55131, Germany; 2 Clinic and Policlinic for Dermatology and Venereology, University Medical Center Rostock, Rostock 18057, Germany; 3 Center for Integrated Protein Science at the Department of Chemistry, Ludwig-Maximilians-Universität München, Munich 81377, Germany

## Abstract

Apurinic/apyrimidinic (AP) sites are a class of highly mutagenic and toxic DNA lesions arising in the genome from a number of exogenous and endogenous sources. Repair of AP lesions takes place predominantly by the base excision pathway (BER). However, among chemically heterogeneous AP lesions formed in DNA, some are resistant to the endonuclease APE1 and thus refractory to BER. Here, we employed two types of reporter constructs accommodating synthetic APE1-resistant AP lesions to investigate the auxiliary repair mechanisms in human cells. By combined analyses of recovery of the transcription rate and suppression of transcriptional mutagenesis at specifically positioned AP lesions, we demonstrate that nucleotide excision repair pathway (NER) efficiently removes BER-resistant AP lesions and significantly enhances the repair of APE1-sensitive ones. Our results further indicate that core NER components XPA and XPF are equally required and that both global genome (GG-NER) and transcription coupled (TC-NER) subpathways contribute to the repair.

## INTRODUCTION

Abasic site is a common name used for DNA damage products formed by loss of nucleobase while retaining the deoxyribose or its fragment with preserved 3′- and 5′-phosphodiester bonds. Such apurinic/apyrimidinic (AP) lesions are constantly generated in DNA by spontaneous hydrolytic reactions and by multiple damage-induced mechanisms (1,2), including enzymatic removal of various nucleobase modifications by DNA glycosylases in the course of base excision repair (BER) (3). Because of their abundance and toxicity, AP sites demand a very efficient repair mechanism. In organisms from bacteria to higher eukaryotes, this is assured primarily by activity of apurinic/apyrimidinic endonucleases, such as human APE1 that generates a hydroxylated 3′-end required for the downstream BER reactions (4). The nuclease activity of APE1 (also annotated as HAP1 and REV1, based on its several known functions) is fundamental for protection of cells from accumulation of endogenously arising abasic DNA lesions (5). Accordingly, the APEX1 gene is essential for early embryonic development in mouse (6–8) and indispensable for growth and survival of human and mouse cell cultures already under physiological DNA damage loads (5,9). Severity of phenotypes caused by the APE1 deficiency thus underscores high toxicity of AP sites and vital importance of the repair.

Most types of AP lesions arising in DNA can assume interconverting constitutions between the furanose and hydroxyaldehyde forms (10,11). It is important to note that the aldehyde at the deoxyribose C1 atom can readily react with nucleophilic groups, leading to strand breakage (12–15) or adduct formation with proteins (16–21), DNA (22–25) and small molecules (12,26,27). Repair of such structurally heterogeneous secondary lesions is challenging, because some of them cannot be efficiently processed by human APE1 (28–30). Since APE1-resistant AP lesions are expected to arise under physiological conditions in the cellular milieu, an additional repair mechanism would be required to protect cells from their toxicity. Based on previous biochemical and genetic evidence, the mechanism in question could be nucleotide excision repair (NER). Thus, Uvr ABC complex of *Escherichia coli* as well as human NER competent cell extracts efficiently recognize and cleave several types of AP lesions (31,32). Furthermore, synergistic phenotypes of mutants with combined BER and NER defects in bacteria (33) and yeast (34–37) indicate that the two pathways functionally overlap during *in vivo* processing of various BER substrates, most likely at the AP site step (34). However, *in vivo* evidence that NER contributes to repair of AP lesions remains largely indirect and it is not known whether some of the mechanisms proposed for unicellular organisms also apply to mammalian cells.

Poor chemical stability of AP sites and their sensitivity to a number of ubiquitous endonucleases, along with the lack of tools for direct specific generation of AP sites in chromosomal DNA, have been the major obstacles to comprehensive characterization of pathways, which would complement BER during repair of AP lesions in cells. Another serious hindrance is the essential nature and diversity of functions of mammalian APE1. To overcome these difficulties, we here employed synthetic BER-resistant AP lesions. We generated reporter vectors harboring such lesions at defined nucleotide positions and employed two independent gene reactivation principles to directly assess contribution of NER to repair.

## MATERIALS AND METHODS

### Cell lines

Immortalized human skin fibroblasts from patients with mutations in the specified nucleotide excision repair genes were obtained from the NIGMS Human Genetic Cell Repository, Coriell Institute for Medical Research (Camden, New Jersey, USA). The XP-A cell line XP20S (GM04312) was analysed along with the matched isogenic cell line (GM15876) complemented with XPA cDNA (38). The CS-B cell line was CS1ANps3g2 (GM16095), the CS-A cell line was CS3BEs3gl (GM16094) and the XP-C cell line was XP4PA-SV-EB (GM15983). The foetal lung fibroblast cell line MRC-5 VA1 (AG10076) was used as repair-proficient reference. The XPF deficient human cell line XPF KO was generated by targeted gene disruption in MRC-5 cells by the CRISPR/Cas9 gene knockout approach (39). The XPF KO cells were analysed in parallel with the corresponding isogenic control designated MRC-5 Vi.

### Reporter constructs for detection of transcription blockage

Reporter constructs containing the specified modifications in the 5′-untranslated region of the enhanced green fluorescent protein (EGFP) gene were generated as described previously (40). Expression vector pZAJ-5C was nicked at tandem sites with the Nb.BsrDI endonuclease (NEB GmbH, Frankfurt am Main, Germany) to generate a 18-nt gap in the transcribed strand followed by annealing and ligation of the specified synthetic oligonucleotides. All oligonucleotides were HPLC-purified and validated by mass spectrometry. Synthetic 18-mer oligonucleotides 5′-CATTGCTTC[THF/S-THF]CTAGCACG containing a tetrahydrofuran AP lesion with either phosphodiester (THF) or phosphorothioate (S-THF) 5′-linkage were from BioSpring GmbH (Frankfurt am Main, Germany). Unmodified reference deoxyribo-oligonucleotide 5′-CATTGCTTCGCTAGCACG was from Eurofins Genomics (Ebersberg, Germany). The oligonucleotide 5′-CATTGC[dT<>dT]CGCTAGCACG containing TT dimer was from TriLink BioTechnologies (San Diego, CA). The oligonucleotide 5′-CATTGCTTC[dG(*N^2^*)-AAF]CTAGCACG containing the 3-(deoxyguanosin- *N^2^*-yl)-2-acetylaminofluorene adduct was produced as described previously (40). The oligonucleotide 5′-CATTGCTTCGC[Fluorescein-dT]AGCACG containing C5-fluorescein-dT adduct was from BioSpring GmbH.

### Reporter constructs for detection of transcriptional mutagenesis

Vector pEGFP_Q205* was generated from the previously described pZAJ vector encoding for functional EGFP protein (41) by introducing the c.613C>T point mutation, which results in the expression of a non-fluorescent EGFP 1-204 fragment. Site-specific incorporation of AP lesions at the nucleotide 613 of the protein coding sequence was accomplished with the help of the Nb.Bpu10I nicking endonuclease (Thermo Fisher Scientific Inc., St. Leon-Rot, Germany), as described previously (42). A 18-nt gap generated in the non-coding (transcribed) DNA strand was used to accommodate synthetic oligonucleotides 5′-TCAGGGCGGACT[THF/S-THF]GGTGC containing the specified AP lesions (BioSpring GmbH) or the respective 5′-TCAGGGCGGACTAGGTGC unmodified oligonucleotide (Eurofins Genomics). The original pZAJ vector accommodating the 5′-TCAGGGCGGACTGGGTGC synthetic oligonucleotide (42) was used as a positive reference for EGFP fluorescence.

### Verification of lesion incorporation into reporter vectors

**Figure 1.**
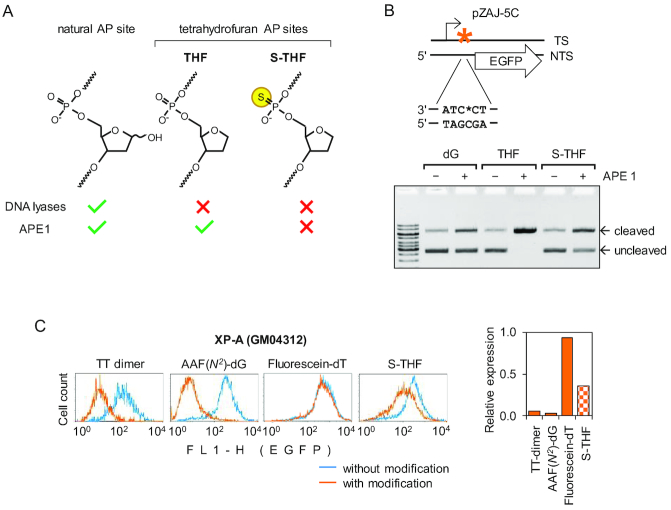
Impairment of transcription by BER-resistant AP lesion positioned at a specific nucleotide in the transcribed strand of the EGFP gene. (**A**) Structures of synthetic tetrahydrofuran (THF and S-THF) AP lesions and reactivity of BER enzymes towards the specified types of AP sites. (**B**) Characterization of reporter constructs containing deoxyguanine (dG) or the specified types of AP lesion at a defined nucleotide (*) in the transcribed DNA strand (TS). Scheme shows position for incorporation of synthetic oligonucleotides containing dG, THF or S-THF with respect to EGFP coding sequence (arrow) and transcription start (broken arrow). To demonstrate the presence of AP lesion, the obtained constructs were incubated with excess of APE1 and analysed by gel electrophoresis in the presence of ethidium bromide. See also Supplementary Figure S1 for more detail. (**C**) Flow cytometry analyses of expression of constructs containing specified modifications in transfected XP-A (GM04312) cells (a representative experiment). EGFP fluorescence distribution plots show expression data overlaid pairwise for each modification and the respective control constructs without modification. Bar chart on the right shows quantification of the EGFP expression, relative to the matched control constructs without the modifications.

Incorporation of synthetic oligonucleotides was monitored as described previously by the formation of covalently closed circular DNA ligation products and concomitant inhibition of ligation in analytic aliquots, in which the oligonucleotide phosphorylation step was omitted (42). Incorporation of thymine dimer was further specifically verified by incision with T4 endonuclease V; the presence of dG(*N^2^*)-AAF and Fluorescein-dT adducts was confirmed by inhibition of cleavage by NheI restriction endonuclease (NEB) at the specific 5′-GCTAGC sequence, as described previously (40). The presence of AP lesions was verified by incubation of 100 ng covalently closed circular DNA constructs with 0.65 units human APE1 (NEB) in the 1 × NEB4 buffer for 1 h at 37°C. Reactions were followed by heat-inactivation for 20 min at 65°C and electrophoresis in agarose gels containing 0.5 mg/l ethidium bromide. Reporter vector preparations used for transfections and the gene expression analyses contained more than 85% of vector DNA in the covalently closed circular form (Figure 1 and Supplementary Figure S1).

### Transfections and gene expression analyses

On the day before transfection cells were plated at 360 000 cells/well on Nunc™ six-well plates in DMEM high glucose medium supplemented with 10% foetal bovine serum (all materials from Thermo Fisher Scientific). Exponentially growing cells were co-transfected with 400 ng per well of the EGFP reporter constructs (with or without the specified modifications) mixed with equal amount of the tracer pDsRed-Monomer-N1 vector (Clontech, Saint-Germain-en-Laye, France) using the Effectene transfection reagent (QIAGEN, Hilden, Germany). At 24 h post transfection, cells were fixed for quantitative determination of EGFP expression by flow cytometry using FACSCalibur™ and the CellQuest™ Pro software (Beckton Dickinson GmbH, Heidelberg, Germany), as described in detail previously (43). In brief, after exclusion of fragmented and aggregated cells by FSC/SSC gating, the DsRed signal (FL2-H) was applied as an additional gating marker for effectively transfected cells. After exclusion of untransfected cells, EGFP fluorescence (FL1-H) distribution plots were generated and average EGFP expression per cell determined as the median of the distribution. The obtained values were used to calculate relative expression levels of constructs with specified modifications based on the reference EGFP construct (generated by incorporation of synthetic oligonucleotide without modifications, transfected in parallel at the same time and measured at the same instrument settings).

## RESULTS

### Generation of a defined BER-resistant AP lesion

Property of DNA lesions to block transcriptional elongation by RNA polymerase II is useful for determination of NER activity based on the capacity of cells to recover transcription of damaged DNA. Accordingly, NER of a number of structurally defined DNA lesions was efficiently measured by a host cell reactivation (HCR) principle, using reporter constructs harbouring a stretch of synthetic DNA with a lesion in the transcribed strand of a reporter gene (40,44,45). To adjust the HCR approach towards specific measurement of NER of abasic sites, it was necessary to exclude the AP site lyase and endonuclease activities associated with the concurrent BER pathway. Because of the multiplicity of proteins with AP site lyase function and the essential character of human APE1 in human cells, a genetic knockout approach cannot be applied to fully inactivate BER. Therefore, our strategy was to generate a BER-resistant apurinic lesion at a unique position in the transcribed DNA strand of a reporter expression construct (Figure 1A and B). To produce such a lesion, we used a chemically stable 2-hydroxymethyl-3-hydroxytetrahydrofuran (THF) building block, which is a close structural 1,2-dideoxy analogue of the most frequently occurring (>99%) cyclic furanose form of the natural 2-deoxyribose AP sites (11). Because of the absent C1 hydroxyl group, THF is resistant to β-lyases but remains an excellent substrate of APE1, in agreement with its structural equivalence to natural AP sites (46,47). Importantly, APE1 endonucleolytic cleavage at the THF lesion can be prevented *in vitro* and *in vivo* by sulfurization of the 5′ phosphodiester bond (46,48). Therefore, we further substituted the linkage 5′ to THF with a phosphorothioate (subsequently referred to as S-THF lesion). As expected, the presence of the phosphorothioate markedly inhibited strand cleavage by purified human APE1 in the circular plasmid DNA (Figure 1B), whereas the THF substrate was fully converted into the nicked circular form at the equivalent APE1 concentrations. This clearly indicates that the THF lesions were efficiently introduced into practically all plasmid molecules by site-specific ligation of the synthetic oligonucleotides and implies that the S-THF AP lesion introduced into the reporter vector is resistant to BER (see also Supplementary Figure S1 for a quantitative comparison of the APE1 activities towards the THF and S-THF substrates). A minor degree of the non-specific nicking of constructs harbouring unmodified oligonucleotide (designated ‘dG’ or ‘dA’) is normal and attributable to cleavage of AP sites, inherently present in plasmid DNA isolated from bacteria.

### BER-resistant AP lesion hinders transcription

Human XP20S (GM04312) cells derived from an XP-A patient have no detectable NER activity (38). Thus, to determine whether the BER-resistant AP lesion in the 5′-untranslated region (UTR) of the EGFP gene elicits transcription block in the absence of repair, we transfected the obtained reporter construct into XP-A cells. In parallel, we analysed analogous constructs containing bona fide transcription blocking DNA lesions TT dimer and the dG(*N^2^*)-AAF adduct (40). Yet another bulky DNA modification analysed was fluorescein-dT adduct, whose transcription blocking potential was unknown. Quantitative analyses of the EGFP expression in transfected cells showed that transcription was inhibited by a factor of 2.5 by a single S-THF lesion in the transcribed strand of the gene as compared to the control construct containing deoxyguanosine in the same position (Figure 1C). Since phosphorothioate bonds by themselves do not hinder gene expression (48), the observed impairment of transcription should be attributed to the presence of unrepaired AP site. As expected, both dG(*N^2^*)-AAF at the same nucleotide and TT-dimer at an adjacent position in the 5′UTR abrogated the EGFP expression almost completely. Of further note is that bulky fluorescein-dT adduct did not cause any significant decrease of gene expression. Comparison between the S-THF and fluorescein-dT lesions thus suggests that there is no direct relationship between the adduct size and the impediment to transcription in cells. Because the impact of abasic site on the EGFP expression is much weaker than the effects of TT dimer and dG(*N^2^*)-AAF, we assume that AP site does not inflict a permanent blockage to transcribing RNA polymerase II; however, it clearly results in a marked decrease of the elongation rate as compared to undamaged DNA. Such a mechanism would be consistent with a ‘slow bypass’ mode, previously reported for yeast RNA polymerase II under reconstituted transcription conditions (49).

### XPA promotes removal of transcription blocking AP lesions

**Figure 2.**
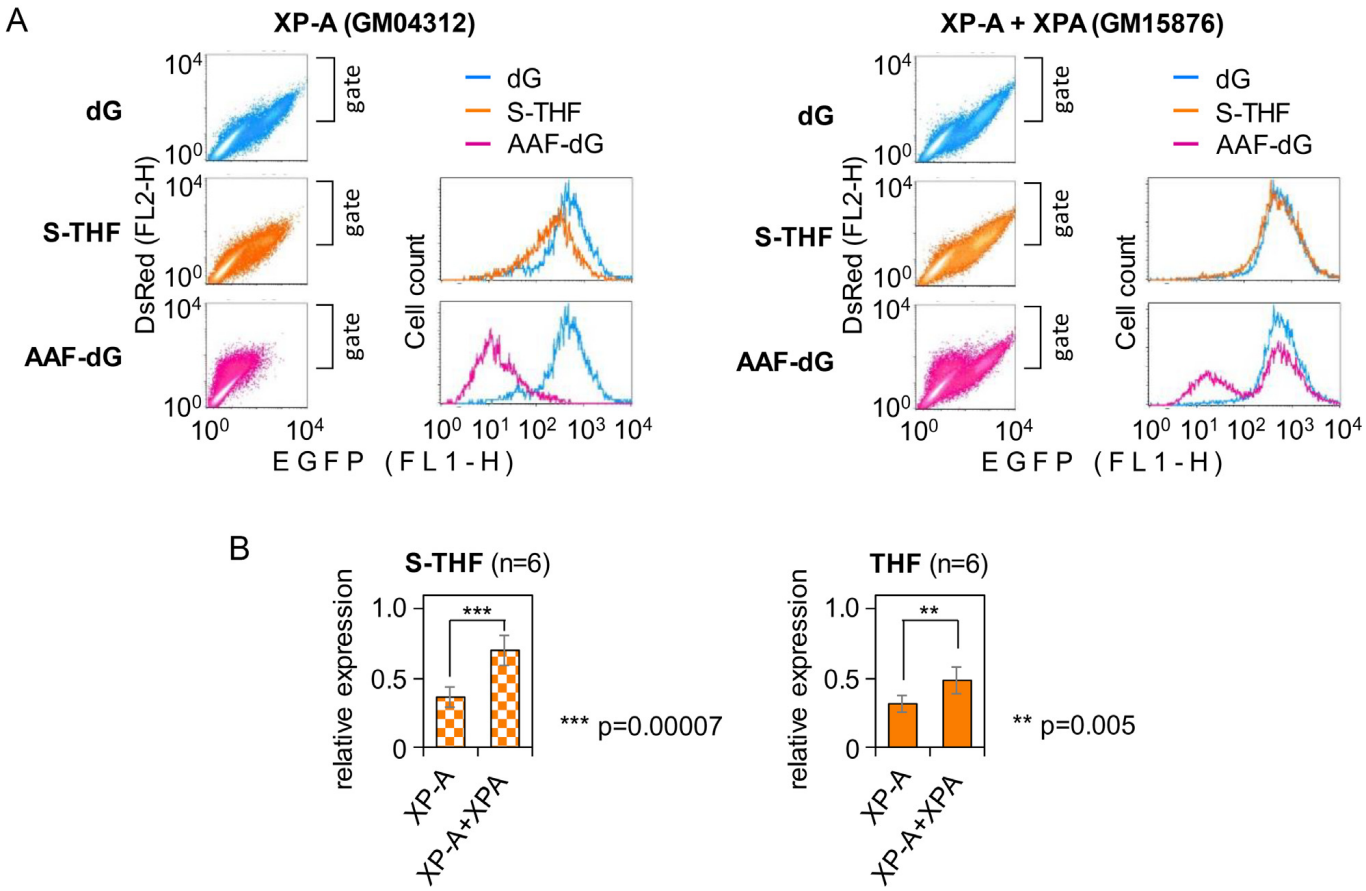
Reactivation of expression constructs containing AP lesions by complementation with XPA. (**A**) Flow cytometry expression analyses of constructs containing dG (blue colour), S-THF (amber) or AAF(*N^2^*)-dG (rose) at the analysed position in transcribed DNA strand of the EGFP gene. Fluorescence scatter plots show co-expression of EGFP with DsRed (as a marker for transfected cells). Cells were gated by DsRed expression to generate fluorescence distribution plots, which show S-THF and AAF(*N^2^*)-dG samples overlaid with a common dG reference sample. (**B**) Quantification of expression of constructs containing the specified AP lesions (S-THF or THF), relative to the dG reference (mean of six independent experiments ± SD; *P*-values calculated by the Student’s *t*-test). See also Supplementary Figure S2.

Complementation with a functional XPA gene restores NER capacity in XP-A cells (38). Hence, to determine whether NER can remove transcription blocking AP lesions, we next assessed the expression of reporter constructs containing synthetic S-THF and dG(*N^2^*)-AAF lesions in the available isogenic cell line complemented with XPA cDNA. In the XPA-complemented (GM15876) cells, we observed two populations with different levels of expression of the reporter construct carrying the dG(*N^2^*)-AAF adduct (Figure 2A). The great part of transfected cells displayed a very efficient recovery of gene expression, which clearly indicates that XPA complementation rescued the NER capacity. The presence of a smaller cell population with persistent NER defect suggests that this fraction of cells did not express XPA. Importantly, complementation with functional XPA gene also resulted in a very efficient reactivation of the construct carrying the BER-resistant AP lesion S-THF (Figure 2A). Because inhibition of transcription by S-THF is much weaker than in the case of dG(*N^2^*)-AAF, the fluorescence distribution analyses could not resolve the minor NER-deficient cell population in this case. Nevertheless, even based on the median overall fluorescence, the effect of XPA was highly significant, as confirmed by six independent experiments. Moreover, reactivation of constructs containing AP lesion with normal 5′ phosphodiester bond (THF) was also significantly improved by XPA complementation (Figure 2B and Supplementary Figure S2), despite susceptibility of the latter lesion to APE1 (Figure 1B). In the case of the APE1 substrate (THF), the recovery of gene expression was somewhat less pronounced than for the BER-resistant AP lesion (S-THF). We previously showed in various cell models that a pre-existing nick (50,51) or nicks generated by APE1 next to various BER substrates—including the THF lesion (48,52,53)—induce transcriptional silencing of reporter transgenes. Under the conditions of complementation with a functional XPA gene, the strand scission by APE1 takes place concurrently to NER activity, which results in a less complete reactivation of the THF than of the S-THF reporter construct (Figure 2B). In summary, the results show that XPA significantly improves repair of AP lesions both in the absence and in the presence of BER.

### XPF and XPC contribute to repair of AP lesions

**Figure 3.**
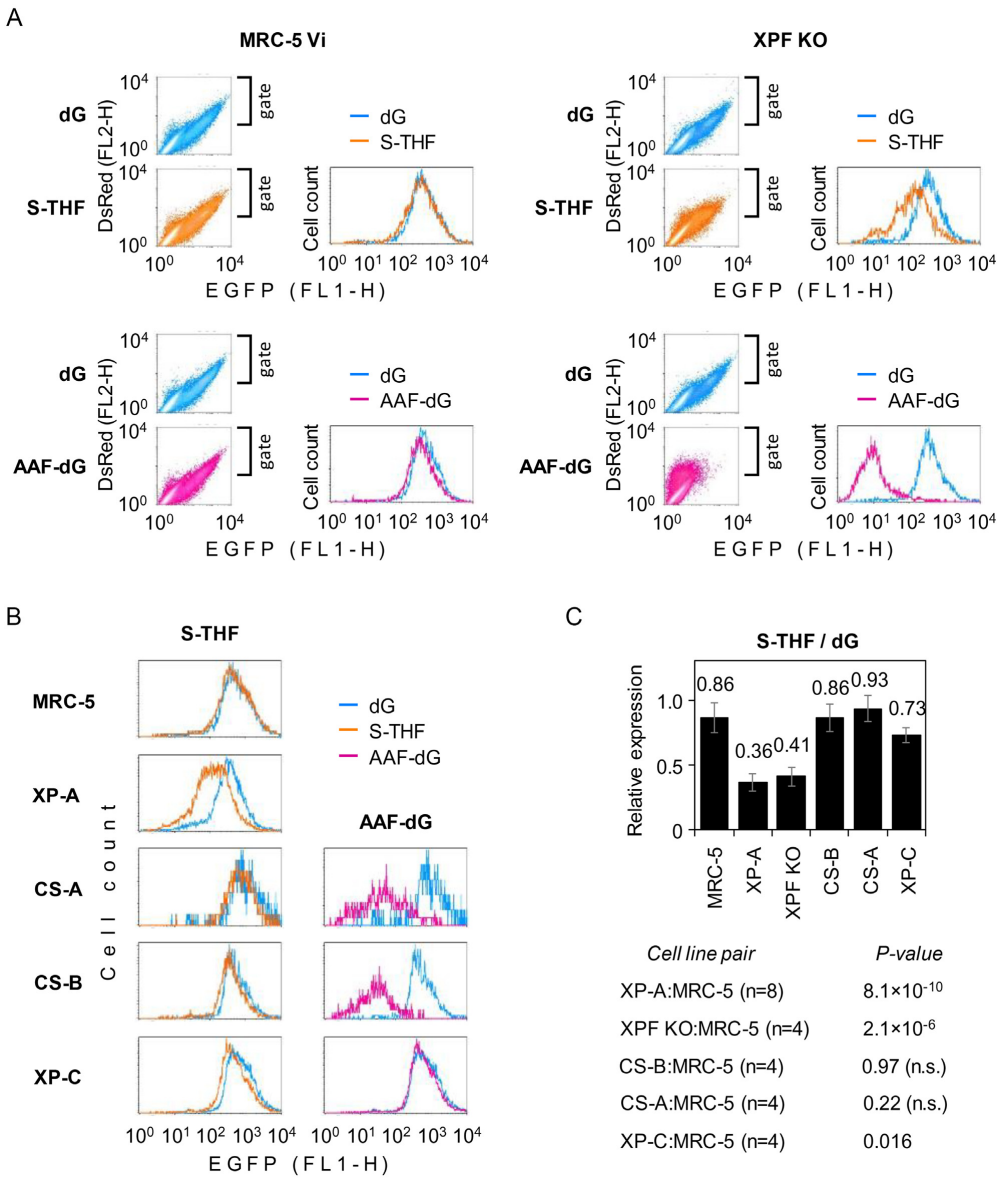
Impact of defects in different NER genes on the removal of the transcription-blocking S-THF lesion. (**A**) HCR of the expression constructs containing S-THF or the AAF(*N^2^*)-dG adduct in the XPF KO and the isogenic MRC-5 Vi cells. EGFP versus DsRed scatter plots and the derived EGFP fluorescence distribution plots (overlaid with the respective control ‘dG’ constructs). (**B**) HCR of the expression construct containing S-THF in human skin fibroblast cell lines of the specified NER complementation groups. Overlaid fluorescent distribution plots were generated as described above but scatter plots were omitted for clarity of presentation. (**C**) Quantification of expression of constructs containing the S-THF AP lesion relative to the dG reference (mean of *n* independent experiments ± SD; *P*-values calculated by the Student’s *t*-test). The MRC-5 value shows pulled data for two independent clones (see ‘Material and Methods’ section).

To verify that the observed role of XPA in the repair of AP sites specifically reflects the NER function, we asked whether a defect in another core NER component would also result in impaired processing of AP lesions. We performed expression analyses of the reporter construct containing S-THF in unrelated NER deficient XPF knockout (XPF KO) cell line (39). The results confirmed a marked impairment of transcription by the AP lesion and revealed a clearly decreased HCR capacity in the XPF KO cells as compared to the maternal MRC-5 Vi cell line (Figure 3A). The absence of NER in the XPF KO cells as well as NER proficiency of the MRC-5 Vi was confirmed by analyses of analogous constructs containing bulky dG(*N^2^*)-AAF adduct. Based on the observation that disruption of the XPF gene closely recapitulates the phenotype of XP-A cells, we attribute impaired processing of the AP lesion in both cell models to the NER deficiency.

Considering that two distinct damage recognition mechanisms operate in the global genome (GG-NER) and the transcription coupled (TC-NER) branches of NER, we further questioned which of the two NER subpathways accounts for the recognition of AP lesions. Because previous reports suggested that TC-NER contributes to processing of AP lesions in yeast (37,54) and since we earlier found that AP lesion has a considerable transcription blocking capacity (Figure 1), we assessed HCR of constructs containing single S-THF lesion in TC-NER deficient cells derived from patients of two different complementation groups (CS-A and CS-B). In parallel, we analysed constructs carrying dG(*N^2^*)-AAF adduct at the same nucleotide position as a reference DNA lesion that is efficiently repaired by TC-NER but cannot be recognized by human GG-NER (40). Surprisingly, the impact of the AP lesion on the gene expression in CS-A and CS-B cell lines was as mild as in fully NER proficient MRC-5 cells (Figure 3B and C), even though the TC-NER deficiency was clearly confirmed in both cell lines by severely impaired HCR of the dG(*N^2^*)-AAF reporter construct (Figure 3B). In the XP-C cells, we observed a somewhat stronger impact of the S-THF lesion on the EGFP gene expression—despite a very efficient TC-NER of the dG(*N^2^*)-AAF adduct in this cell line. Even if it was minor in the magnitude, this effect was reproducible and statistically significant (Figure 3C), thus suggesting that recognition of abasic lesions is slightly compromised in the absence of GG-NER. Nevertheless, judging by sensitivity to the AP lesion, we conclude that none of the mutations that selectively affect either TC-NER or GG-NER damage recognition pathway yields a phenotype equivalent to a total NER deficiency (as in XPA- or XPF-null cells). Functional CSA and CSB genes are both essential for TC-NER and so is XPC for GG-NER. Thus, unless we hypothesize the existence of a hitherto unknown third damage recognition principle within NER, it is reasonable to assume that both GG-NER and TC-NER can initiate removal of transcription blocking AP lesions from DNA.

As an additional point, we want to stress that, despite the different genetic background of cell models used, the defects in the XPA and XPF genes resulted in quantitatively identical functional outcomes of the AP lesion in the HCR assay (Figure 3C). Considering that both XPA and XPF have essential functions in the NER pathway, such a result exactly meets expectations for a bona fide NER substrate. Thus, reactivation of reporter constructs containing the reference NER substrate dG(*N^2^*)-AAF also equally required XPA and XPF (Figures 2A and 3A).

### Transcriptional bypass of AP site results in mutant RNA

**Figure 4.**
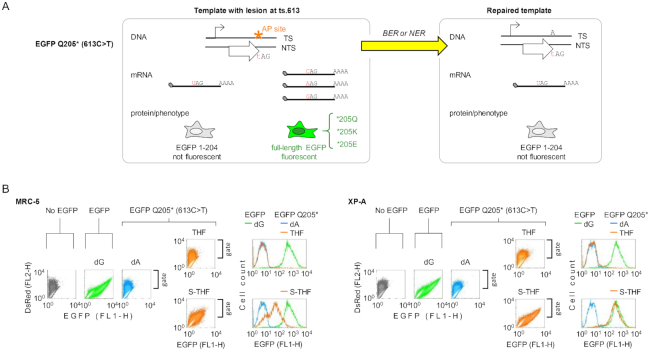
Transcriptional mutagenesis at the BER-resistant abasic site in the template DNA and its suppression by NER. (**A**) Scheme of the reporter for detection of ribonucleotide misincorporation opposite to AP-lesion in the template DNA. Substitution of 613U in mRNA to any other ribonucleotide results in reversion to a fluorescent EGFP. (**B**) Flow cytometry assay for detection of the mRNA single nucleotide substitutions induced by the specified AP lesions (THF, S-THF) in the MRC-5 (group of panels on the left) and XP-A (group of panels on the right) cell lines. Fluorescence scatter plots show full data for individual samples from a representative experiment. The derived EGFP fluorescence distribution plots show overlaid data for EGFP construct without modification (green colour) and EGFP Q205* constructs without modification (blue) or with the indicated lesion (amber). The nature of the nucleotide/modification in the template DNA strand is indicated above the plots. Note the right shift of S-THF plots compared to dA.

Considering that GG-NER and TC-NER both can initiate repair of AP lesions in transcribed DNA, it would be interesting to know which of the two damage recognition pathways dominates in human cells. However, because the reporter gene reactivation in cells with selective defects in either GG-NER or TC-NER closely approached the levels observed in fully NER proficient host cells, we could not judge about relative efficiencies of GG-NER and TC-NER in quantitative terms (Figure 3C). Seeking to improve the sensitivity, we generated expression constructs with single AP lesion placed at varying distances from the transcription start in a hope to achieve a more pronounced impairment of transcriptional elongation at some position. However, we observed only slight variation of the degree of transcriptional blockage by AP lesions at different positions (data not shown). We then decided to make use of the high miscoding potential of abasic lesions, which led us to construction of a positive readout reporter for detection of ribonucleotide misincorporation at the AP lesion site during transcription (Figure 4A). Such RNA polymerase errors at the damage sites are widely termed as transcriptional mutagenesis (55). Because of the lack of consensus in the literature about the nature of ribonucleotide preferentially incorporated by human RNA polymerase II opposite to AP lesions in the DNA template (56,57), we were aiming at a reporter system in which any misincorporated ribonucleotide would result in a reversal to a fluorescent EGFP. From the analysed single nucleotide substitutions leading to the non-fluorescent phenotype, we selected the c.613C>T chain termination mutant encoding for a truncated EGFP Q205* protein, because we found in a phenotypic screen that subsequent substitutions of 613A in the transcribed DNA strand for any other nucleotide efficiently restored the EGFP fluorescence (Figure 4A and Supplementary Figure S3).

To specifically incorporate synthetic AP lesions at the nucleotide 613 of the identified EGFP Q205* mutant, we used tandem Nb.Bpu10I sites intrinsically available in the transcribed DNA strand of the EGFP gene (40). Generation and biochemical characterization of the EGFP Q205* constructs containing synthetic AP lesions (THF or S-THF) at the nucleotide 613 is shown in Supplementary Figure S3. Expression analyses of the EGFP Q205* construct containing a single BER-resistant AP lesion (S-THF) at the nucleotide 613 revealed a pronounced gain of EGFP fluorescence in transfected cells in comparison with a reference construct containing dA, thus indicating that transcriptional bypass of the AP lesion is accompanied with a high error rate (Figure 4B). The reversal to the fluorescent EGFP phenotype was clearly observed in MRC-5 cells (the group of panels on the left) and further greatly enhanced in NER deficient XP-A cells (the group of panels on the right). In the XP-A cells, this corresponded to at least 60-fold increase of the median EGFP fluorescence intensity over the background expression of the reference dA construct. In contrast, the AP lesion susceptible to APE1 (THF) did not lead to re-gain of the EGFP fluorescence, thus indicating that transcriptional mutagenesis was efficiently prevented by BER. In summary, the results imply that BER of AP lesions is normally accomplished within a very short time, which does not allow synthesis of significant amounts of mutant mRNA (as in the case of THF). However, if BER is compromised (as in the case of S-THF), prolonged persistence of AP lesions leads to phenotypically relevant accumulation of mutant transcripts. The results further show that the proportion of mutant mRNA becomes overwhelming if both BER and NER are unavailable.

### Multiple NER pathway components protect from transcriptional mutagenesis at AP sites

**Figure 5.**
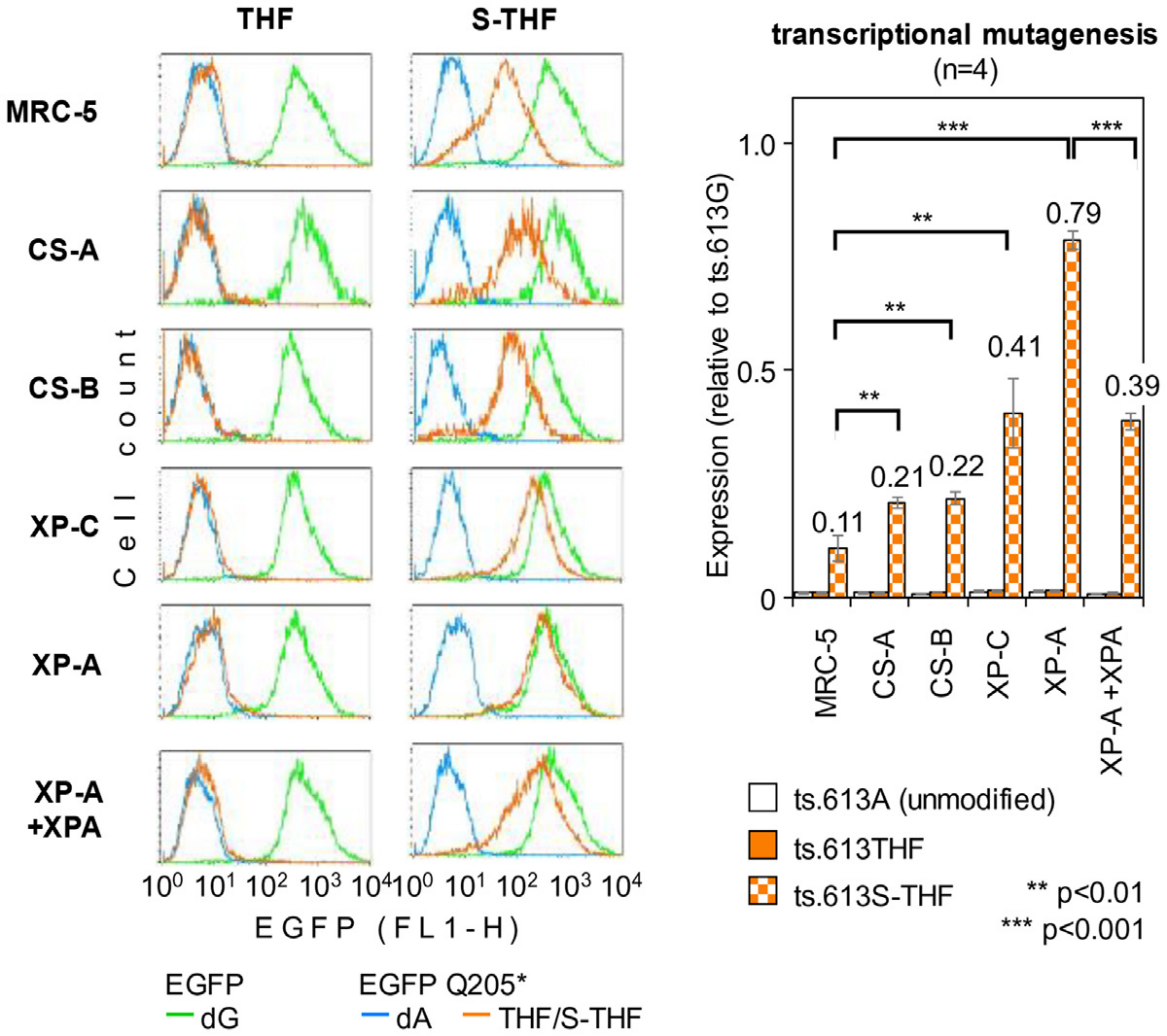
Transcriptional mutagenesis by THF and S-THF lesions in the panel of NER deficient cell lines: overlaid fluorescence distribution plots from a representative experiment and a bar chart showing quantification of the EGFP expression of the specified pEGFP Q205* constructs, relative to the original EGFP without modification in the transcribed DNA strand (ts.613G). Data of four independent experiments (mean ±SD; *P*-values calculated by the Student's *t*-test).

Highly efficient reversion to the fluorescent phenotype inflicted by erroneous transcriptional bypass of unrepaired AP site now provided a sufficiently broad dynamic range for determination of specific contributions of the TC-NER and GG-NER subpathways to repair of AP lesions, based on the inverse relationship between NER capacity of host cells and the resulting EGFP fluorescence intensity. We therefore measured the gain of EGFP fluorescence in cell lines derived from patients of different NER complementation groups following transfections with the EGFP 613C>T reporter constructs containing THF or S-THF at the nucleotide 613 of the transcribed DNA strand (Figure 5). We observed that the presence of the APE1-sensitive THF lesion did not induce any significant increase of the EGFP fluorescent signal compared to the reference construct containing dA at the same position, which is in agreement with efficient BER of this type of AP lesion in all cell lines. In contrast, S-THF lesion caused strong increase of the EGFP fluorescence in all cell lines tested, thus indicating significant levels of ribonucleotide misincorporation at the lesion site. Furthermore, these levels were measurably increased in all NER complementation groups as compared to NER proficient MRC-5 cell line. Thus, based on median EGFP fluorescence intensity in transfected cells, a defect in the TC-NER pathway doubled the frequency of reversion to functional EGFP, no matter whether the affected gene was CSA or CSB. Inactivation of the GG-NER pathway (in XP-C cells) caused an even stronger (4-fold) increase of the transcriptional mutagenesis rate. The strongest (almost 8-fold) gain of the EGFP signal was documented in the XP-A cell line, in which both GG-NER and TC-NER are not functional. On the other hand, complementation with functional XPA gene significantly reduced the EGFP fluorescence intensity in the XP-A cell line, which proves that restoration of the NER function counteracts the transcriptional mutagenesis. Taken together, the results indicate that both GG-NER and, to a lesser extent, TC-NER account for repair of the mutagenic AP lesion in human cells.

## DISCUSSION

Specific analyses of repair of AP sites in mammalian cells are technically challenging. Most common treatments used to generate AP lesions in the genome DNA of mammalian cells are alkylating substances and ionizing radiation. Because these agents have complex damage spectra and since the resulting lesions (including AP sites) are chemically labile, it is not possible to assign the observed effects strictly to AP lesions. As an alternative approach, AP lesions can be generated in a controlled way in DNA of external origin and subsequently delivered to cells to investigate the repair. A recent report proposed a host cell reactivation setup to determine variations of BER capacity between human cell lines towards synthetic AP sites introduced at specific nucleotide positions in DNA (57). An important advantage of such experimental system is the possibility to use chemically stable AP lesions in order to prevent their decay and uncontrolled reactivity, once delivered to cells. Among several AP site analogues characterized, THF has the closest structural similarity to naturally occurring AP sites (11). Because of the lack of the aldehyde group on C1 atom, THF in DNA cannot undergo enzymatic β-elimination (Supplementary Figure S4) and is essentially deprived of chemical reactivity under physiological conditions. At the same time, it remains an excellent substrate for APE1 (47,58) as well as ideal model for structural studies (59). Importantly, THF lesions can be further modified by sulfurization of the 5′ phosphodiester linkage to render them resistant to APE1 (46,58) without causing a significant structural alteration of the DNA helix (59). Here, we used these properties to generate reporters suited for analyses of alternative repair mechanisms in human cells with unaltered APE1 function. Combination of the constructs carrying THF with APE1-resistant (phosphorothioate) and APE1-sensitive (phosphodiester) 5′ linkages gave us possibility to compare functional outcomes of AP lesions in the absence and in the presence of BER in genetically and physiologically unperturbed cells.

Using two independent gene reactivation principles, we revealed important molecular details of toxicity of AP sites in transcribed DNA. By analysing outcomes of the BER-resistant AP lesion in cell lines with critical NER defects (XP-A, XPF KO), we documented mild impairment of transcription efficiency by the lesion positioned in a non-coding region of the transcribed DNA strand (Figure 1) and confirmed high miscoding potential of the AP lesion during transcription (Figure 4). Both ability of THF to block transcription and its capacity to induce transcriptional mutagenesis are overall in agreement with results reported by Samson *et al.* (57); however, the lesion used by others was not resistant to APE1 and possible contribution of NER to the repair was not considered at the time of previous publication. It seems that the degree of transcription blockage by THF might have been greatly overestimated formerly and it may be useful to adjust interpretation of the available quantitative data considering our new findings.

Further, and most importantly, we obtained clear evidence of contribution of NER to repair of AP lesions in human cells. We showed in two pairs of isogenic cell lines with opposite NER statuses that NER rescued the expression of constructs containing transcription blocking AP lesions (Figures 2 and 3). Independently, using a different reporter system, we found that NER deficiency leads to massive increase of transcriptional mutagenesis specifically at the AP site (Figure 4). Both effects are most strongly pronounced at the APE1-resistant AP lesions. The very high (up to 80%) rate of reversal to the fluorescent EGFP phenotype by transcriptional mutagenesis in XP-A cells (Figure 4) corroborates the assumption that S-THF lesion is resistant to BER in cells. Taken together, the results strongly suggest that NER is an important backup pathway for the repair of AP lesions under the conditions when the 5′ endonucleolytic cleavage cannot take place. Extrapolated to physiological conditions of DNA damage, this result means that NER could be of vital importance for the repair of subclasses of APE1-resistant AP lesions, such as deoxyribose rests whose C1 atom is reduced or covalently bound to a stable chemical group (28–30).

Differently from S-THF, the APE1-sensitive THF lesion did not induce transcriptional mutagenesis; however, the XPA complementation significantly reduced the degree of transcriptional impairment in its presence (Figure 2). The results thus imply that NER activity parallels or complements BER also at the APE1-sensitive AP lesion by removing some transcription blocking structure. In the case of THF, this could be either the AP lesion itself or its strand-cleaved product generated by APE1.

Based on the recovery of gene expression levels, we deduce that both GG-NER and TC-NER can recognize AP lesions in transcribed DNA (Figure 3C). Furthermore, using the levels of transcriptional mutagenesis at the nucleotide opposite to the BER-resistant AP lesion as a marker for NER impairment, we can derive quantitative estimates for the fractions of AP lesions processed by each of the NER subpathways. Assuming a constant RNA polymerase error rate at the AP lesion with the likelihood of incorporation of a wrong ribonucleotide smaller than 1, the doubled rates of reversal to a fluorescent EGFP in the Cockayne syndrome (CS-A and CS-B) cell lines should reflect at least a 2-fold decrease in the repair efficiency in the absence of TC-NER (Figure 5). Proportionally, the 4-fold gain of EGFP fluorescence in the XP-C cell line indicates an even more profound impairment of repair in the absence of GG-NER. Ultimately, further increase of the phenotypic mutation rate in the XP-A cell line to almost 80% clearly indicates a severe impairment or even a complete absence of repair.

Although not reported to date in human systems, contribution of NER to repair of AP lesions is not entirely unexpected. Ample genetic and functional data strongly suggested a protective role of NER against depurination damage in yeast (34–37), pointing in particular to the roles of the TC-NER pathway components (37,54,60). However, extrapolation of these conclusions onto human NER needs caution. According to current view, the TC-NER damage recognition mechanism is initiated by arrest of an elongating RNA polymerase complex at the lesion (61). It is uncertain whether AP lesions have a transcription blocking capacity to a degree necessary for the TC-NER activation. Even though RNA synthesis by mammalian RNA polymerase II is robustly interrupted by ‘natural’ deoxyribose AP lesions (62,63), the likely cause of the observed premature termination of transcription was either enzymatic or chemical decay of this very labile AP lesion in the template DNA (62). In contrast, structurally analogous stable THF lesions can be bypassed with rather high efficiencies by human RNA polymerase II (56) and induce merely a transient stalling of the yeast Pol II (49). Arguably, the observed kinetic barrier could be sufficient to launch TC-NER (49). Our observation of some residual NER capacity towards BER-resistant AP lesion in the GG-NER deficient XP-C cells (Figure 5) in principle supports this view. Nevertheless, as far as it can be judged from current results (Figures 3C and 5), TC-NER is not the predominant pathway in human cells and AP lesions can be processed even more efficiently by GG-NER. It is thus intriguing to propose that NER would likely act also on AP lesions in the non-transcribed DNA strand and elsewhere throughout the genome. Unfortunately, we could not test this presumption in our reporter system, because S-THF lesion in the non-transcribed DNA strand did not interfere with the gene expression to a significant extent (data not shown).

The finding that NER contributes to repair of AP lesion may have great importance for understanding of biological outcomes of this type of DNA damage. The estimated rate of spontaneous generation of AP lesions in the human cell genome exceeds 10 000/day and this load can be greatly increased under exposure to exogenous damaging agents (64). Depending on the nature of the damaging agent, a broad spectrum of structurally related but chemically diverse AP lesions can arise in DNA, which include so-called ‘classic’ AP sites generated by chemical or enzymatic hydrolysis of the N-glycosydic bond and various species of oxidized AP lesions arising from reactions of deoxyribose with free radicals (10,65). Not all forms of AP lesions are efficiently processed by BER and some undergo chemical reactions leading to BER-resistant damage, which would have been deleterious if there were no backup repair mechanism. Xeroderma pigmentosum (XP) patients with impaired NER often suffer from neurological conditions and manifest degenerative features that have not been clearly linked to the classical NER substrates. Considering that AP sites commonly arise spontaneously or as intermediate products during BER of multiple types of DNA lesions (including endogenously generated damage), there is a possibility that impaired processing of AP lesions contributes to the clinical features of XP.

## Supplementary Material

gkz558_Supplemental_File
