# Supplementary material for: Nucleotide excision repair of abasic DNA lesions
Source: Nucleic Acids Res. 2019 Jun 21;47(16):8537–47. doi: 10.1093/nar/gkz558 (PMC6895268; doi:10.1093/nar/gkz558)
Supplement: gkz558_Supplemental_File [file gkz558_supplemental_file.pdf]

Nucleotide excision repair of abasic DNA lesions

Nataliya Kitsera, Marta Rodriguez-Alvarez, Steffen Emmert, Thomas Carell and Andriy Khobta

Supplementary information:

- Supplementary Figure S1
- Supplementary Figure S2
- Supplementary Figure S3
- Supplementary Figure S4

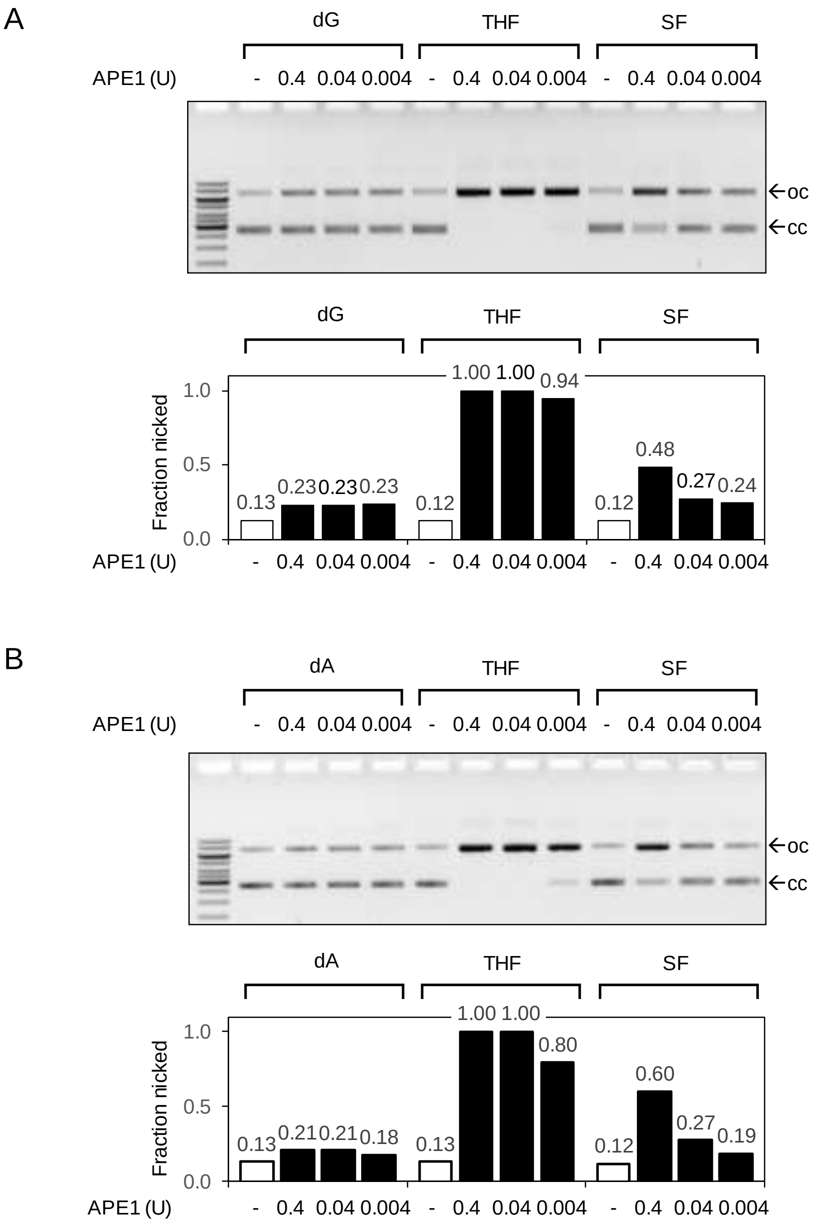

Supplementary Figure S1. Quantitative analyses of APE1 activity towards the THF and S-THF AP lesions incorporated into the reporter vectors used for analyses of (A) transcriptional blockage (pZAJ-5C) and (B) transcriptional mutagenesis (pEGFP_Q205*). The constructs containing the specified types of AP lesion at the defined positions (as specified in Materials and Methods) were incubated with a range of APE1 concentrations. Reactions containing 50 ng vector DNA were incubated 30 minutes at 37°C in 7.5 ul 1× NEB4 buffer supplemented with 0.2 mg/ml bovine serum albumin, followed by heat-inactivation. APE1 activity was measured by conversion of the covalently closed DNA (cc) into the open circular form (oc), as determined by gel electrophoresis in the presence of ethidium bromide and band densitometry. Relative amounts of DNA in bands were calculated by applying a coefficient of 2.4 to correct for a lower ethidium bromide binding capacity of the topologically closed plasmid form, as described previously (48). The results show that >100 times higher APE1 concentrations are required to cleave the S-THF substrates at levels equivalent to the cleavage of the control THF substrates.

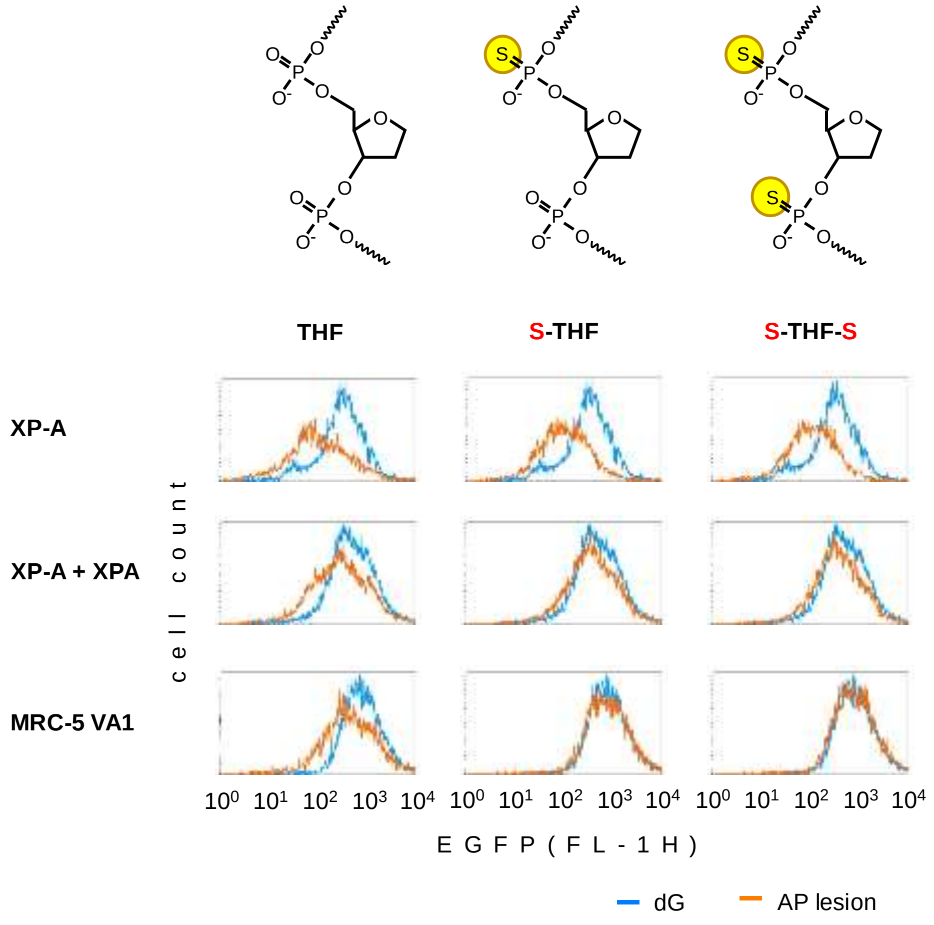

Supplementary Figure S2. Representative HCR data of expression constructs containing THF with sulfurized linkages at the indicated positions. Flow cytometry fluorescence distribution plots of transfected XP-A, the isogenic XPA-complemented (XP-A + XPA), and unrelated NER-proficient MRC-5 VA1 cells were generated as in Figure 2A. The APE1-resistant AP lesions (S-THF and S-THF-S) negatively affect gene expression only in XP-A cells, because AP lesions are efficiently removed in the NER proficient cell lines. The decrease of transcription induced by the THF lesion (with unmodified phosphodiester bonding) is a cumulative effect of the strand cleaved APE1 product (manifested in all cell lines) and transcription blockage by THF itself (visible as an additional decline of the EGFP signal in the XP-A cell line). The observed effects are highly reproducible and significant (Figure 2B).

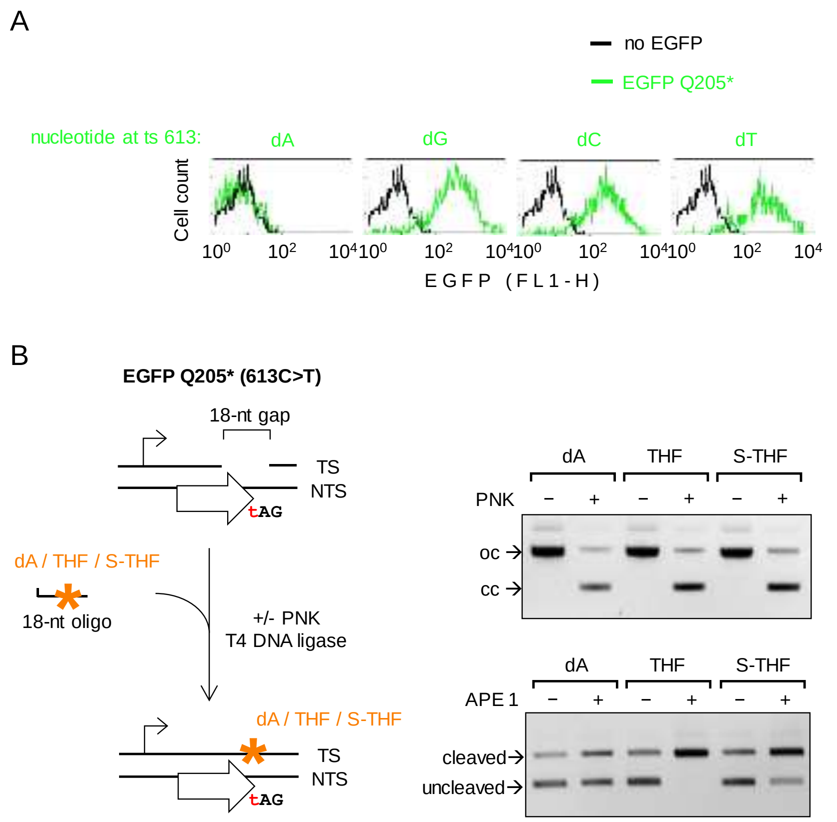

Supplementary Figure S3. Generation and characterisation of the EGFP Q205* constructs containing synthetic AP lesions (THF or S-THF) at nucleotide ts.613. (A) Phenotype of constructs containing different nucleotides at the specified position in the transcribed DNA strand (ts.613). The EGFP c.613C>T nonsense mutation results in a non-fluorescent protein. Oligonucleotides containing dA/dG/dC/dT at the ts.613 were ligated into a gap generated by Nb.Bpu10I in the transcribed DNA strand, followed by transfection into DLD1 cells for the EGFP expression analyses. All nucleotides, except dA, exhibit bright EGFP fluorescence signal. (B) Scheme of incorporation of synthetic oligonucleotides containing dA or the specified modifications into the 18-nucleotide gap in the transcribed DNA strand. Only a fragment of circular vector DNA containing the EGFP gene is shown. Persistence of the open circular (oc) form of plasmid DNA after ligation in the absence of polynucleotide kinase (PNK) and generation of the covalently closed form (cc) in the presence of PNK confirm efficient incorporation of heterologous synthetic oligonucleotides. Efficient cleavage of THF and only partial cleavage of S-THF by human APE1. Indicated covalently closed constructs were incubated with excess of APE1 and analysed by gel electrophoresis in the presence of ethidium bromide.

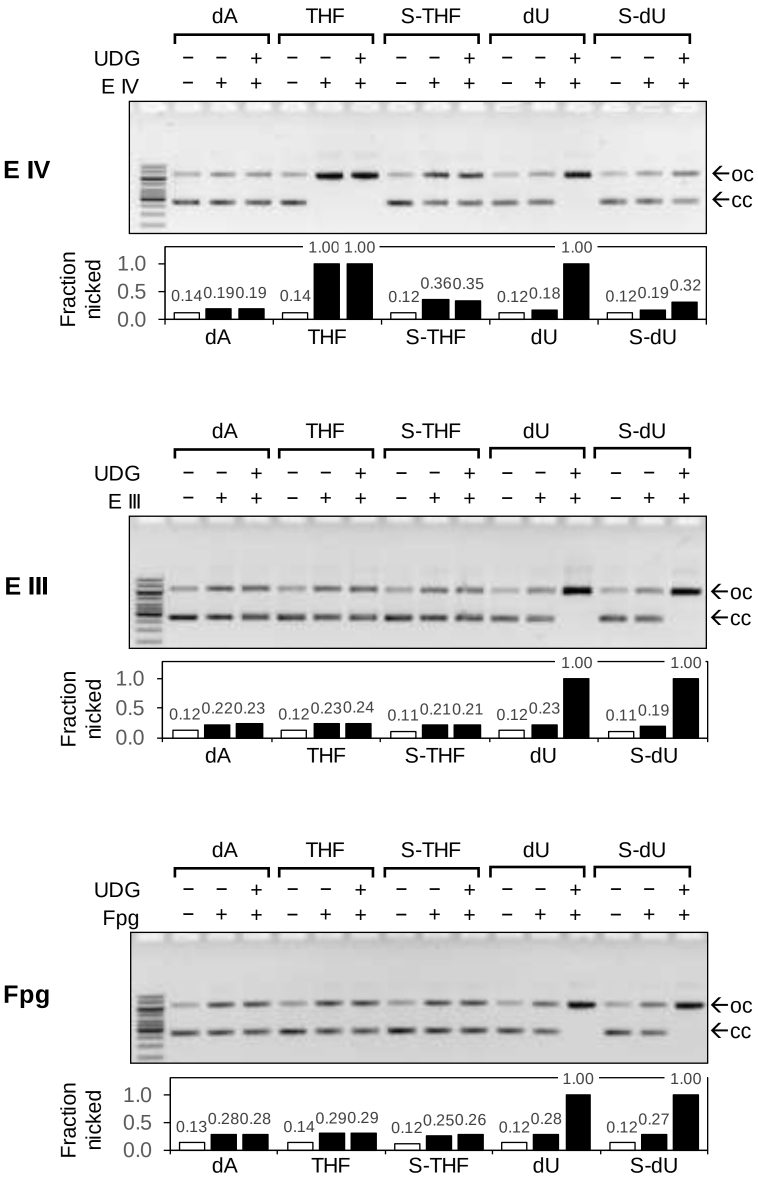

Supplementary Figure S4. Characterisation of activities of DNA lyases towards the indicated types of synthetic (THF, S-THF) and "classic" natural AP lesions in the covalently closed vector constructs. To generate "classic" AP lesions, vector constructs containing dU and s-dU were treated with the specific DNA glycosylase UDG at the saturating conditions. All enzymes were purchased from NEB. All enzymes were added at 2 U/100 ng DNA and incubated 1 hour at 37°C under conditions described previously (42). As expected, both THF and the "classic" AP lesion (but not their "S-" counterparts with sulfurized linkages) are efficiently cleaved by Endonuclease IV (E IV), which is a class II AP endonuclease with a catalytic mechanism analogous to human APE1. Importantly, THF and S-THF are completely resistant to strand cleavage by the beta-lyases Endonuclease III (E III) and Fpg – in contrast to the "natural" AP lesions derived from dU and S-dU.
